# Clinicopathological Features of Frontal Fibrosing Alopecia (FFA) in 26 females: A Retrospective Study

**DOI:** 10.30699/ijp.2024.2014122.3194

**Published:** 2024-07-24

**Authors:** Maryam Khalili, Nafise Esmaeilpour, Simin Shamsi-Meymandi, Rezvan Amiri, Fatemeh Gheisoori, Mahin Aflatoonian

**Affiliations:** 1 Department of Dermatology, Clinical Research Development Unit, Afzalipour Hospital, Kerman University of Medical Sciences, Kerman, Iran; 2 Kerman University of Medical Sciences, Kerman, Iran; 3 Department of Dermatology, Pathology and Stem Cells Research Center, Afzalipour School of Medicine, Kerman University of Medical Sciences, Kerman, Iran; 4 Department of Dermatology, Leishmaniasis Research Center, Kerman University of Medical Sciences, Kerman, Iran

**Keywords:** Cicatricial alopecia, Frontal fibrosing alopecia, Hair loss, Patholog

## Abstract

**Background & Objective:**

FFA usually has a gradual subtle course and might be overlooked by physicians or misdiagnosed with other types of hair loss including androgenetic alopecia, traction alopecia, and other types of patterned alopecia. In this study, we described clinicopathological features of patients with FFA referring for skin biopsy.

**Methods:**

This is a retrospective cross-sectional study on 26 patients with a diagnosis of FFA based on clinicopathological features. Firstly, the demographic and clinical features of patients were extracted from an electronic database. Then, skin biopsy specimens were reviewed regarding the presence or absence of hair follicles, site and severity of infiltrations, and presence of fibrosis.

**Results:**

Most of the patients were over fifty years of age (57.7%) with a mean age of 50.73 ± 10.03 years. Frontal region involvement was observed in all of the cases. Eyebrow hair loss was observed in 38.5% of cases. The most frequent clinical findings were the absence of vellus hairs in frontotemporal regions (96.2%) and perifollicular erythema (92.3%). The most common pathological features were involvement of the vellus hairs (84.6%), replacement of follicular epithelium with fibrous sheath (80.8%), and destruction of sebaceous glands (69.2%). Peri-infundibular and peri-bulbar interface changes were observed in 50% and 61.5% of skin biopsies, respectively. Perifollicular fibrosis was demonstrated in half of the skin biopsies.

**Conclusion:**

FFA is most commonly observed in females after the 5th decade of life. The absence of vellus hairs and the replacement of follicular epithelium with fibrous sheath are the most common clinical and pathological features of the disease, respectively.

## Introduction

FFA is a cicatricial alopecia that is considered a unique subtype of lichen planopillaris (LPP). It most commonly affects postmenopausal women. However, there are some reports of involvement of younger women and men. Currently, different factors such as genetic predisposition, stressful events, autoimmunity (associated with hypothyroidism, vitiligo, lichen sclerosus et atrophicus and Sjögren syndrome), hormonal (hyperandrogenic state) and environmental factors (sun exposure, smoking, cosmetic material, and allergens) have been proposed in the pathogenesis of FFA(1-6). 

Classically, FFA presents gradual and symmetric regression of hairline in frontotemporal areas and eyebrow hair loss. It can also occasionally involve eyelashes as well as hair follicles of other parts of the scalp (including occipital and parietal regions) or body surfaces. In comparison to other types of hair loss, it has more preference to involve vellus hairs than terminal hairs (7-10).

FFA usually has a gradual subtle course; thus, it is not generally noticed by patients until extensive recession of the anterior hairline happens. On the other hand, it might be overlooked by physicians or misdiagnosed with other types of hair loss including androgenetic alopecia, traction alopecia, and other types of patterned alopecia (11-15). Clinicopathological features have been reported in only a few studies in Iran; in this study, we described clinicopathological features of patients with FFA referring for skin biopsy.

## Material and Methods

This is a retrospective cross-sectional study on 26 patients with a diagnosis of referred for skin biopsy between January 2018 and May 2023. Firstly, demographic features (age and sex) and clinical characteristics of the lesions including site and duration of alopecia, type of the lesions (including the presence of perifollicular erythema, hyperkeratosis, hyperpigmented macules, facial papules, and absence of vellus hairs) and symptoms were extracted from the electronic database from dermatopathology department of our center. Then, skin biopsies which were performed by a 4-mm punch were reviewed by two fellowships of dermatopathology. Skin biopsies were evaluated in both horizontal and vertical sections. Details of pathological features of the lesions including interface changes, involvement of sebaceous glands and terminal hair follicles, characteristics of infiltrations (site and severity of inflammatory cells) as well as and severity of fibrosis were evaluated. This proposal was approved by our ethical committee with the ethical code of IR.KMU.AH.REC.1400.293.

**Statistical Analysis:**

SPSS 22 (IBM, Armonk, NY, USA) was utilized for the analysis of data. Prevalence, frequency, and mean (± standard deviation) were used for the description of data. Chi-square and independent t-tests were applied to assess the association between the final diagnosis and qualitative and quantitative variables, respectively.

## Results

**Fig. 1:**
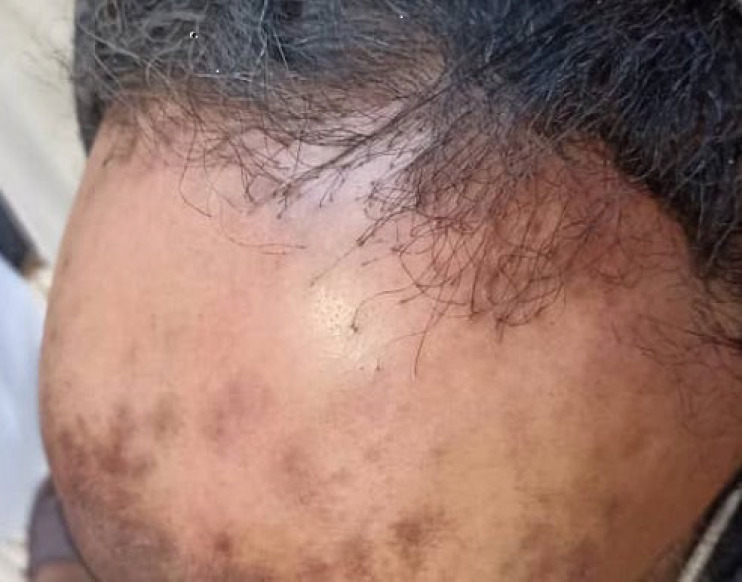
A 45-year-old female with regression of the frontotemporal hairline, absence of the vellus hairs, perifollicular accentuation, and hyperpigmented patches in the frontal region.

In this study, we evaluated 26 females with FFA referred for skin biopsy. Most of the patients were over 50 years old (57.7%) with a mean age of 50.73±10.03 (ranging from 35 to 67) years (Table 1). The mean duration of hair loss was 25.3 (ranges 3-60) months. Frontal involvement was observed in all of the cases. Moreover, temporal, parietal, and occipital areas were involved in 38.5%, 7.7%, and 7.7% of the cases, respectively. Eyebrow hair loss was observed along with scalp hair loss in 38.5% of the cases. The most frequent clinical findings were the absence of the vellus hairs in frontotemporal regions (96.2%) and perifollicular erythema (92.3%). Symptoms including pruritus and burning sensation were observed in 57.7% of the cases (Table 1, Figure 1). 

**Fig. 2:**
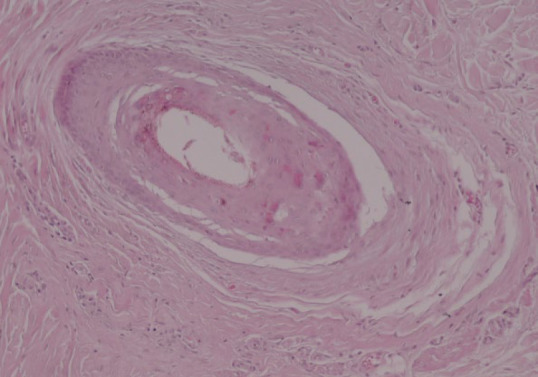
Intra-epithelium apoptotic cells, onion-like perifollicular concentric fibrosis, and mild perifollicular infiltration (hematoxylin and eosin staining, ×400 magnification).

**Fig. 3:**
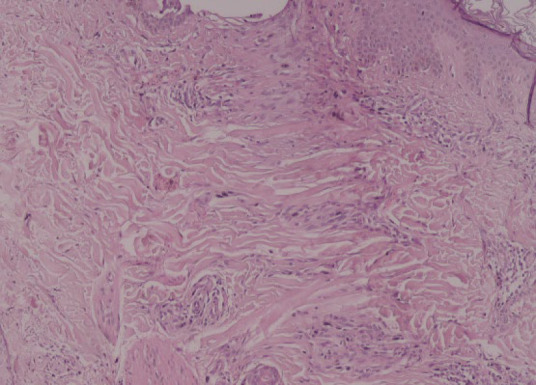
Fibrous sheath replacement of the follicular epithelium (hematoxylin and eosin staining, ×400 magnification).

**Fig. 4:**
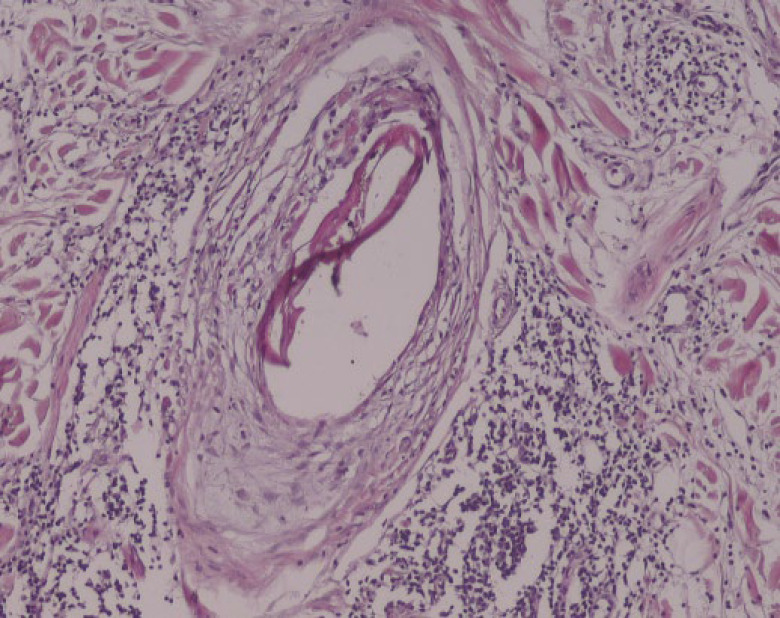
Premature desquamation of the inner root sheath, severe perifollicular infiltration of lymphohistocytic cells and moderate dermal fibrosis (hematoxylin and eosin staining, ×400 magnification).

The most common pathological features were involvement of the vellus hairs (84.6%), replacement of the terminal hairs with fibrous sheaths (80.8%), and destruction of the sebaceous glands (69.2%). Peri-infundibular and peri-bulbar interface changes were observed in 50% and 61.5% of skin biopsies, respectively. Peri-bulbar inflammation which involved both vellus and terminal hair follicles was observed in 53.8% of cases. Inflammation was mild-to-moderate in most of the cases (92.8%). Moreover, 42.3% of the cases had exocytosis of inflammatory cells within the epithelium of the hair follicles which was mostly within less than 1/3 of the thickness of the follicular epithelium (63.6%). No interfollicular interface changes or peri-eccrine inflammation was demonstrated in the skin biopsies. Peri-bulbar and per-infundibular fibrosis were demonstrated in 38.5% and 11.5% of the skin biopsies. Furthermore, intradermal fibrosis was observed in all of the cases, which was more prominent in 42.3% of the cases. (Table 2, Figures 2, 3, and 4).

**Table 1:** Demographic and clinical features of the patients with frontal fibrosing alopecia

Variables		
Age, Year, Mean ± SD (min-max)		50.73 ± 10.03 (35-67)
Sex, N (%)	Female	26 (100)
Duration, month, Mean ± SD (min, max)		25.30 ± 19.64 (3-60)
Site N (%)	Frontal	26 (100)
	Temporal	10 (38.5)
	Others	4 (15.4)
Eyebrow involvement, N (%)		10 (38.5)
Perifollicular erythema, N (%)		24 (92.3)
Perifollicular hyperkeratosis, N (%)		13 (50)
Perifollicular scaling, N (%)		13 (50)
Lack of vellus hair, N (%)		25 (96.2)
Diffuse reticular erythematous pattern, N (%)		2 (7.7)
Hyperpigmented macules, N (%)		8 (30.8)
Symptom, N (%)	Pruritus	8 (30.8)
	Burning sensation	7 (26.9)
	No symptom	11 (42.3)
Facial papules, N (%)	Yes	5 (19.2)

**Table 2:** Pathological features of the patients with frontal fibrosing alopecia

Pathological features			Frequency (%)
Epidermal atrophy			5 (19.2)
Interfollicular interface changes			0 (0)
Peri-infundibular interface changes			13 (50)
Peri-bulbar interface change			16(61.5)
Peri-infundibular concentric fibrosis			3 (11.5)
Peri-bulbar concentric fibrosis			10 (38.5)
Sebaceous glands destruction			18 (69.2)
Replacement of hair follicles with fibrous tracts			21 (80.8)
Premature desquamation of the inner root sheath			3 (11.5)
Necrotic keratinocytes within follicular epithelium			12 (46.2)
Increased ratio of terminal catagen-telogen hairs			5 (19.2)
Involvement of vellus			22 (84.6)
Peri-bulbar inflammation	Severity	Mild	6 (23.1)
		Moderate	7 (26.9)
		Severe	1 (3.8)
	Exocytosis	Exocytosis < 1/3 of the thickness of epithelium	7 (26.9)
		Exocytosis > 1/3 of the thickness of epithelium	4 (15.4)
Inflammation within the eccrine gland			0 (0)
Dermal infiltration		Perivascular	15 (57.7)
		Interstitial	13 (50)
Dermal fibrosis		Mild	15 (57.7)
		Marked	11 (42.3)

## Discussion

FFA predominantly involves females, and it can be asymptomatic; however, sometimes symptoms including itching, burning sensation, pain, and tenderness are reported by patients. Clinically, alopecic areas devoid of follicular orifices with a lack of vellus hairs, and the presence of perifollicular erythema are evident. The absence of clinical characteristics of sun exposure in involved frontotemporal areas is another distinctive feature of this type of alopecia that presents as whitish-pink-colored areas. Moreover, perifollicular accentuation as a result of hyperkeratosis of hair follicles is another key clinical feature. In addition, keratosis-pilaris-like lesions, diffuse erythema in a reticular pattern, and hyperpigmented macules might be found in the facial area (16-24). 

Results of this study demonstrated that all of the FFA patients were female and were older than 30 years old, and most of them were after the fifth decade of life. The mean duration of hair loss was 2.1 years. The most commonly involved regions were frontal and temporal (100% and 38.5%, respectively). Moreover, 38.5% of cases also had involvement of eyebrow hairs. More than half of the cases had symptoms of pruritus or burning sensation (57.7%). The most common clinical features were perifollicular erythema (92.3%) and perifollicular scales/hyperkeratosis (50%). Facial papules and hyperpigmented macules were observed in 19.2% and 30.8% of cases, respectively. 

In one study by Imhof *et al.* on 122 cases with FFA, the mean age of the patients was 62.1 years. The mean duration of the hair loss was 4.7 years. The most common sites of hair loss were frontal (99.3%) and temporal (52%). Eyebrow involvement was reported in 60.1% of the cases. Perifollicular erythema was observed in 87.2% of the cases, while perifollicular hyperkeratosis and scaling were observed in a lower number of the cases (37.8% and 66.2%, respectively). The majority of the cases reported pruritus (67.6%) (20). 

Doche *et al.* in another study, evaluated the clinicopathological features of 28 patients (92.8% female) with FFA. The mean age of the patients was 58.1 years (range of 35-83 years), and the mean duration of the disease was 28 months. Eyebrow hair loss was observed in 75% of the cases. Clinical features such as facial papules and hyperpigmented macules were observed in 28.6% and 21.4% of the cases, respectively (24). 

Pathological evaluation in earlier stages of the FFA demonstrates lichenoid perifollicular infiltration of lymphocytes, necrotic keratinocytes within the outer root sheet of the hair follicles, and onion skin-like perifollicular lamellar fibroplasia. Complete replacement of hair follicles with fibrous sheath tract and destruction of sebaceous glands have been described in the later stages of the disease (23-30). The most common clinicopathological differential diagnoses of FFA are LPP and cutaneous lupus erythematosus (CLE). The absence of infiltration in the interfollicular epidermis, a higher percentage of apoptotic cells within the follicular epithelium, and more tendency to involve vellus hairs can distinguish FFA from LPP (4, 12). Peri-eccrine and perivascular lymphoplasma cell infiltration and mucin deposition in the reticular dermis are pathological features that can differentiate CLE from FFA (23-29). 

In the current study, the most common specific pathological features were involvement of the vellus hairs (84.6%), replacement of terminal hairs with fibrous sheaths (80.8%), and destruction of sebaceous glands (69.2%). Moreover, intra-epithelium apoptotic cells (46.2%) and exocytosis of the inflammatory cells within the follicular epithelium (42.3%) were other pathological characteristics. Peri-infundibular and peribulbar interface changes were observed in 50% and 61.5% of the skin biopsies, respectively. Peri-bulbar inflammation was observed in 53.8% of the cases that had mostly mild-to-moderate severity (92.9%) and involved less than 1/3 of the follicular epithelium. Moreover, no inflammation was observed in the interfollicular epithelium or peri-eccrine areas. Peri-bulbar and peri-infundibular fibrosis were demonstrated in 38.5% and 11.5% of the cases, respectively.

In the study of Imhof *et al.*, the most common pathological characteristics were peri-infundibular inflammation (50.8%), perifollicular fibrosis (36.1%), and perifollicular inflammation (18.9%) (20). In another study by Doche *et al.*, perifollicular inflammation was observed in 89.3% of the cases which was severe only in 3.5% of the cases (21). 

In the study by Gálvez-Canseco *et al.*, clinicopathological features of 44 patients with FFA were evaluated. The most common pathological features were perifollicular involvement of the terminal hairs (93.2%) and destruction of the sebaceous glands (90.9%). In addition, interface vacuolar changes of the follicular epithelium, apoptotic keratinocytes, and exocytosis of lymphocytes within follicular epithelium were observed in 81.8%,72.7%, and 65.9% of the cases, respectively. In the majority of the samples (36.4%), exocytosis of lymphocytes involved more than 1/3 of follicular epithelium thickness (vs. 29.6% with less than 1/3 of epithelium thickness). Perifollicular inflammation was mostly (95.4%) mild-to-moderate (vs. severe inflammation in 4.6%). In contrast, no skin biopsy demonstrated interfollicular inflammation below eccrine glands. Moreover, mucin deposition was only observed in 2.3% of the cases (25).

## Limitations:

This study was designed retrospectively, thus we only had access to clinical and pathological features of the disease activity. Future comprehensive studies that evaluate the correlation of dermoscopic features with clinicopathological features and the impact of them on the response of patients to treatment would be recommended.

## Conclusion

In this study, all of the patients with FFA were females; mostly after the fifth decade of life. Most of the patients had hair loss for more than two years before diagnosis. Frontotemporal regions were the most common sites of involvement. The most frequent clinical findings were the absence of the vellus hairs, perifollicular erythema, and perifollicular hyperkeratosis/scaling. The most common pathological features were loss of vellus hairs, replacement of terminal hairs with fibrous sheath, and destruction of the sebaceous glands. 
